# Oral high dose vitamin D for the treatment of diabetic patients with COVID-19: A protocol for systematic review and meta-analysis

**DOI:** 10.1097/MD.0000000000024517

**Published:** 2021-03-05

**Authors:** Xiaoya Nie, Jiaoxue Chen, Fang Ye, Hui Wang, Liang Tang, Lang Wang

**Affiliations:** a Department of Special demand ward; b Department of metabolic Endocrinology, Zhuzhou Central Hospital, Zhuzhou, Hunan, China.

**Keywords:** corona virus disease 2019, diabetes, meta-analysis, protocol, systematic review, vitamin D

## Abstract

**Background:**

Type 2 diabetes mellitus patients complicated with infections experience severe vitamin D deficiency. High-dose vitamin D is applied to the treatment of corona virus disease 2019 (COVID-19) by some researchers, and good results have been achieved. However, the efficacy of vitamin D in the treatment of infections in COVID-19 patients with diabetes remains unclarified. This study aims to explore the effect of oral high-dose vitamin D in the treatment of diabetic patients with COVID-19.

**Methods:**

Randomized controlled trials about the application of high-dose vitamin D in the treatment of diabetic patients with COVID-19 will be retrieved from such electronic databases as Embase, PubMed, Cochrane Central Register of Controlled Trials, China National Knowledge Infrastructure database, Chinese Wanfang database and Chinese Biomedical Literature database. The retrieval time is from their inception to December 2020. According to the pre-designed inclusion/exclusion criteria, the data will be extracted independently by two researchers. The risk of bias of the included studies will be assessed by the Cochrane collaboration's tool. Meta-analysis will be conducted by using Revman 5.3 software.

**Results:**

A high-quality and comprehensive evaluation of oral high-dose vitamin D for the treatment of diabetic patients with COVID-19 will be made.

**Conclusion:**

The article will provide more convincing evidence and evidence-based guidance for clinical practice.

**Ethics and dissemination:**

The private information of individuals will not be made public, and this systematic evaluation will also not infringe on the rights of participants. Ethical approval is not required. Research results may be published in a peer-reviewed journal or disseminated in relevant conferences.

**PROSPERO Registration Number:**

CRD42020214284.

## 1 Introduction

Patients with type 2 diabetes are susceptible to all kinds of viruses and at a high risk of acute and chronic infections due to immune changes.^[1,2,3]^ In December 2019, a novel β-coronavirus called Corona Virus Disease 2019 (COVID-19) broke out in Wuhan, China. It has the characteristics of rapid mutation, multiple hosts and strong infectivity.^[4]^ Therefore, diabetic patients are extremely vulnerable to COVID-19 infection, and it is more difficult for diabetic patients with COVID-19 infection to be restored to health.^[5,6]^

In recent years, vitamin D has been frequently applied to the treatment of diabetes. For type 2 diabetes mellitus patients complicated with infections with a severe lack of vitamin D, vitamin D supplementation can effectively alleviate the extent of infections.^[7,8]^ Ohaegbulam's study showed that activating the vitamin D receptor expressed in immune cells could directly reduce the secretion of inflammatory cytokines (such as interleukin-6) and indirectly affect C-reactive protein.^[9]^ Oral vitamin D in patients with type 2 diabetes can improve their immune function and lower the incidence of infections.^[10]^ High-dose vitamin D has been employed to treat COVID-19 by some researchers,^[9]^ but the therapeutic effect of vitamin D for diabetic patients infected with COVID-19 needs to be elucidated.

Given the fact that the efficacy of high-dose vitamin D in COVID-19 patients with diabetes lacks the support of high-quality evidence, a systematic evaluation of this treatment will be carried out, which is expected to provide evidence-based guidance for clinical applications.

## 2 Materials and methods

This study follows the Preferred Reporting Items for Systematic Reviews and Meta-analysis Protocols statement guidelines.^[14]^ It has been registered at PROSPERO with a registration number of CRD42020214284.

### 2.1 Selection criteria

#### 2.1.1 Type of studies

Randomized controlled trials that investigate the efficacy of high-dose vitamin D in the treatment of diabetic patients with COVID-19 will be enrolled in this study.

#### 2.1.2 Types of patients

Inclusion criteria:

(1) Diabetes mellitus patients diagnosed with COVID-19;
(2) Patients aged 18 years old or above;
(3) Patients with a Mini-mental State Examination score > 21;
(4) Patients with the blood ketone value < 0.4 mol/L.

Exclusion criteria:

(1) Patients with renal failure;
(2) Patients with moderate or severe heart disease;
(3) Patients with moderate to severe liver failure.

#### 2.1.3 Types of interventions and comparison

The treatment group will be given high dose vitamin D (50000IU) once a day. The control group will receive routine treatment, or small dose vitamin D (1000 IU) once a day.

#### 2.1.4 Types of outcomes

(1) Routine blood test (including RBC, Hb, WBC, PLT, etc.);
(2) C-reactive protein;
(3) Serum amyloid A;
(4) Length of stay;
(5) Activities of daily living;
(6) Quality of Life.

### 2.2 Exclusion criteria

(1) Duplicate publications;
(2) Articles without full text access;
(3) Studies whose literature forms are abstracts, conference papers or graduation theses.
(4) Studies with extremely low quality.

### 2.3 Search strategy

Studies published from the database inception to December 2020 will be collected from Embase, PubMed, Cochrane Central Register of Controlled Trials, China National Knowledge Infrastructure database, Chinese Wanfang database Chinese and Biomedical Literature database. Taking PubMed as an example, the retrieval strategy is shown in Table 1.

**Table 1:** Search strategy in PubMed database.

Number	Search terms
#1	Diabetes[Title/Abstract]
#2	Diabetic [Title/Abstract]
#3	T2DM [Title/Abstract]
#4	Diabetes Mellitus [Mesh]
#5	#1 OR #2 OR #3 OR #4
#6	Corona Virus Disease 2019 [Title/Abstract]
#7	Novel coronavirus [Title/Abstract]
#8	COVID-19 [Mesh]
#9	#6 OR #7 OR #8
#10	VD[Title/Abstract]
#11	Vitamin D [Mesh]
#12	1,25 (OH) 2D3 [Title/Abstract]
#13	25-Hydroxyvitamin D 2 [Mesh]
#14	#10 OR #11 OR #12 OR #13
#15	#5 AND #9 AND #14

### 2.4 Study selection and data extraction

#### 2.4.1 Selection of studies

**Figure 1:**
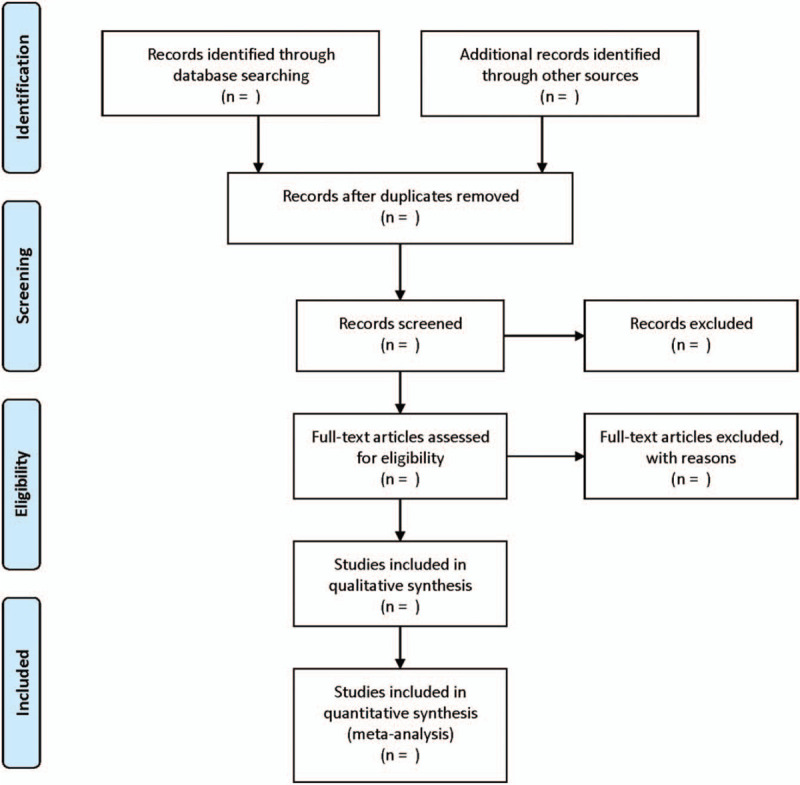
Flow diagram of study selection process.

Two reviewers will search for the studies independently according to the retrieval strategy at first. Then, they will review and screen the studies alone. In case of disagreement, the two reviewers would resolve it together or seek help from other reviewers. The detailed literature selection process is described in Figure 1.

#### 2.4.2 Data extraction

The two reviewers independently extracted and checked the data of studies, including the publication year, publication area, participant characteristics, sample size, age, disease condition, drug dosage, control measures, study cycle, results, adverse events, etc. For missing or unclear data, we will try to contact the corresponding author.

#### 2.4.3 Assessment of the risk of bias

The risk of bias of the included studies will be evaluated by the two reviewers according to the items included in the Cochrane collaboration's tool. Studies will be classified into 3 types according to the assessment results: low-risk, unclear, and high-risk. If there are any differences, the decision would be made through either discussion of two reviewers or consultation with a third-party researcher.

#### 2.4.4 Measurement of the treatment effect

Two categories of variables will be expressed using the risk ratio and its 95% confidence interval, and the mean difference and its 95% confidence intervals will be used for representing continuous variables.

#### 2.4.5 Dealing with missing data

Missing or unclear data will be obtained by contacting the corresponding authors. If it fails, intention analysis would be carried out.

#### 2.4.6 Assessment of heterogeneity

The inter-study heterogeneity will be analyzed by using *Q* test and *I*^2^. When *P* ≥ .1 and *I*^*2*^ ≤ 50%, it shows that there is homogeneity among the studies; when *P* ≥ .1 and *I*^2^ > 50%, it indicates that there is heterogeneity among the studies.

#### 2.4.7 Treatment of heterogeneity

In case of heterogeneity among studies, subgroup analysis or sensitivity analysis would be performed on the factors that may lead to heterogeneity to trace the source of heterogeneity. If there is statistical heterogeneity among the studies but no clinical or methodological heterogeneity, the random effect model would be used.

#### 2.4.8 Data synthesis

Meta-analysis will be conducted by using Revman 5.3 software (The Cochrane Collaboration, Oxford, England). The fixed effect model would be used for meta-analysis in the condition that no statistical heterogeneity exists among the results. The random effect model would be adopted under the circumstance of only statistical heterogeneity found among the results. If the heterogeneity is excessively large and the source of heterogeneity cannot be determined, only descriptive analysis would be conducted.

#### 2.4.9 Assessment of the reporting bias

The publication bias will be detected by the funnel plot when there are ≥10 studies included, or by the fail-safe number when there are <10 studies. The potential publication bias will be quantitatively assessed with the Egger's and Begg's tests.

#### 2.4.10 Subgroup analysis

Subgroup analysis will be performed according to the drug dose (<50000 or ≥ 50000).

#### 2.4.11 Sensitivity analysis

The stability of the meta-analysis results will be measured by a one-by-one elimination approach.

#### 2.4.12 Ethics and dissemination

The content of this article does not involve moral approval or ethical review and would be presented in print or at relevant conferences.

## 3 Discussion

COVID-19 has high infectivity and significant mortality, especially in patients with basic diseases (such as diabetes mellitus).^[11]^ A report in Wuhan revealed that 20% of 41 people newly infected with COVID-19 had diabetes.^[12]^ Another article suggested that 12% of the 140 hospitalized COVID-19-infected patients were diabetic.^[13]^ In addition, it was established by a study that among 173 severely ill patients, 16.2% had diabetes.^[14]^ In China, the mortality in COVID-19 patients with diabetes is about three times higher than the total mortality in patients with COVID-19.^[15]^ Diabetes has been recognized as a risk factor for increased mortality from COVID-19 infection.^[16]^ There is ample evidence of a shared pathophysiologic and mechanistic link between diabetes and COVID-19 infection, especially when the vitamin D level is lower than 10 ng / ml.^[16,17]^ Therefore, vitamin D potentially has good effect in the treatment of diabetic patients with COVID-19.

It is believed that vitamin D possesses a variety of antioxidant and immunomodulatory properties.^[18]^ 1,25 (OH) 2D3 can stimulate the native immune system and inhibit the adaptive immune response.^[19,20]^ Vitamin D receptor is expressed in almost all cells of the immune system.^[16]^ According to Agyun's research, COVID-19 infection impairs multiple organs, while vitamin D supplementation can prevent COVID-19 from injuring organs.^[21]^ As claimed by a study, a dose of vitamin D as high as 100,000 IU (or 50,000 IU) can shorten the length of hospital stay of patients with severe mechanical ventilation, and is also safe for them.^[22]^ COVID-19 patients that have received a high dose of vitamin D supplementation present normalized vitamin D levels and improved clinical sysptoms.^[9]^ Vitamin D deficiency prevails in diabetic patients, so it is necessary for diabetic patients with COVID-19 to supplement high-dose vitamin D.

Due to limited original research findings, it remains a doubt whether high-dose vitamin D can improve the prognosis of diabetic patients with COVID-19, and this issue is most concerned by the medical community at present. To the best of our knowledge, this systematic review is the first one of its kind about the effects of vitamin D on diabetic patients with COVID-19. It should be noted that the lack of adequate randomized controlled trials may be a limitation of this meta-analysis.

## Author contributions

**Data collection**: Xiaoya Nie and Lang Wang.

**Funding support**: Lang Wang.

**Literature retrieval**: Xiaoya Nie and Lang Wang.

**Resources:** Jiaoxue Chen, Fang Ye.

**Software operating**: Xiaoya Nie and Lang Wang.

**Supervision**: Jiaoxue Chen and Fang Ye.

**Writing – original draft**: Hui Wang and Liang Tang.

**Writing – review & editing**: Xiaoya Nie and Lang Wang.
